# Safety of Progestogen Hormonal Contraceptive Methods during Lactation: An Overview

**DOI:** 10.3390/clinpract14030083

**Published:** 2024-06-04

**Authors:** Karolina Chmaj-Wierzchowska, Katarzyna Wszołek, Katarzyna Tomczyk, Maciej Wilczak

**Affiliations:** Department of Maternal and Child Health, Poznan University of Medical Sciences, 61-701 Poznan, Poland; katarzyna.wszolek@ump.edu.pl (K.W.);

**Keywords:** breastfeeding, lactation, contraception

## Abstract

Background: Breastfeeding is a process for not only nourishing infants but also for building a unique emotional bond between mother and child. Therefore, the ideal contraception during lactation should not affect lactation (milk composition, milk volume) and offspring development. Objectives: This study aims to analyze the literature on the safety of progestogen hormonal contraceptive methods during lactation. Methods: We conducted a thorough search across various databases, including the National Library of Medicine (PubMed), and the Cochrane Database, Drugs and Lactation Database (LactMed). Our search utilized specific phrases such as: “lactation” and “breastfeeding” and “oral contraception” with “drospirenone” or “desogestrel”, with “subcutaneous etonogestrel implant” or “etonogestrel implant”, with “levonorgestrel-releasing intrauterine system”, and “emergency contraception”, with “levonorgestrel” or “ulipristal acetate”. Conclusions: Based on published scientific reports, progestogen hormonal contraceptives can be considered a relatively safe solution for women desiring to continue feeding their infant with their milk while using hormonal contraception. It is important to seek guidance on selecting the best contraception method based on the latest medical knowledge, tailored to the individual needs and clinical circumstances of each woman and place of residence. A woman should always be informed of the potential risks of such a treatment and then allowed to make her own decision based on the knowledge received from a specialist.

## 1. Introduction

Breastfeeding is a process not only for nourishing infants but also for building a unique emotional bond between mother and child. The properties of breast milk, which cannot be replaced by any milk substitute mixture produced so far, are also crucial. In addition to its nutritional value, human milk also plays a functional role [1], benefiting all systems and organs of the dynamically developing body of newborns and infants [2], and meeting all nutrient requirements up to the sixth month of a child’s life [3,4,5]. A mother’s own milk is the first choice for feeding premature babies [2,6,7]. Moreover, breastfeeding also acts as a protective factor for mothers, reducing the risk of developing breast cancer, ovarian cancer, type 2 diabetes, and hypertension [8,9,10]. Hence, a breastfeeding woman may face a fundamental dilemma when it comes to using contraceptives, as the choice and timing of contraception can affect not only the breastfeeding process but also the baby’s development. Nonhormonal contraception methods are preferred for breastfeeding women, with progestogen-only methods being another option. Combined hormonal contraceptive methods containing estrogen and progestogen can be considered as a third contraceptive option during lactation [11,12]. However, combined oral contraceptives should not be used in lactating mothers before 42 days postpartum, as the disadvantages of this method generally outweigh the benefits during the first 6 weeks to 6 months postpartum [13,14,15]. This is due to an increased risk of thromboembolic lesions and a potential decrease in milk supply during the first few days of estrogen exposure [16,17]. Additionally, there may be a risk of gynecomastia, mainly in male newborns exposed to very high doses of ethinylestradiol [17,18,19]. Therefore, the ideal contraception during lactation should not affect lactation (milk composition, milk volume) and offspring development. At the same time, it should also be harmless and acceptable to partners, safe and reversible, simple to use, and should not limit the possibility of intercourse or affect libido. Consulting healthcare professionals for personalized contraceptive recommendations adapted to each woman’s clinical situation and individual needs is essential. This study aims to analyze the literature on the safety of progestogen hormonal contraceptive methods during lactation.

## 2. Materials and Methods

We conducted a thorough search across various databases, including the National Library of Medicine (PubMed), and the Cochrane Database. Our search utilized specific phrases such as: “lactation” and “breastfeeding” and “oral contraception” with “drospirenone” or “desogestrel”, with “subcutaneous etonogestrel implant” or “etonogestrel implant”, with “levonorgestrel-releasing intrauterine system”, and “emergency contraception”, with “levonorgestrel” or “ulipristal acetate”. Our search spanned from 1980 to the present date. The articles must be written in English. The inclusion criteria were “Books and Documents”, “Clinical Trial”, “Meta-Analysis”, “Randomized Controlled Trial”, “Review and Systematic Review”. Exclusion criteria were studies in vitro or ex vivo and all animal studies, authors’ and expert opinions, questionnaires and editorials. The search strategy for electronic databases and the results are shown in Table 1. We carefully reviewed the titles and abstracts of the retrieved articles, selecting only those relevant to our research topic, after eliminating duplicates. The final stage was the verification of data in the Drugs and Lactation Database (LactMed). 

## 3. Discussion

### 3.1. Scheduled Single-Ingredient Hormonal Contraception in the Form of Oral Pills

During lactation, depending on the clinical situation and individual needs, women have the option to use single-component hormone therapy with either 75 μg of desogestrel or 4 mg of drospirenone.

#### 3.1.1. Effectiveness

When taking single-ingredient progestational pills, the Pearl index ranges from 0.14 with ideal usage to 0.41 with typical usage [20]. The Pearl index recorded for desogestrel (DSG) is 0.41 under the assumption of consistent intake every 24 ± 3 h. For drospirenone, the Pearl index fluctuates depending on the study, ranging from 0.51 [21] to 0.7258 [22]. The half-life of drospirenone alone (*T* ½ = 30 h) significantly prolongs the efficacy, persisting even if a dose is missed [23,24]. In obese patients <35 years of age, with a BMI ≥ 30 kg/m^2^, the efficacy of single-ingredient contraception with drospirenone might be lower, resulting in a Pearl index of 2.9 [25], yet with a concurrently high level of safety [22,25].

#### 3.1.2. Effects of Contraception on Breastfeeding and the Newborn Baby

There is limited current research on the safety of hormonal contraception and its impact on newborns. A prospective cohort study by Goulding et al. [26] among 1349 women who used contraception 3 months after delivery assessed the relationship between postpartum contraception and breastfeeding. During this period, the women used various contraceptive methods: 53% (*n* = 720) nonhormonal contraceptives, 19% (*n* = 256) combined hormonal contraceptives, 16% (*n* = 217) single-ingredient progestogen-only pills, 7% (*n* = 92) intrauterine therapeutic systems (IUDs) and 5% (*n* = 64) depot medroxyprogesterone acetate. Compared to nonhormonal contraceptives, the adjusted odds ratio (aOR) for single-ingredient contraception was 3.15 (95% CI 1.42–7.02), while for combined contraceptives, it was 0.17 (95% CI 0.10–0.29). The predicted probability of breastfeeding 4 months after delivery was 90% for users of nonhormonal contraceptives, 97% for users of single-ingredient pills, and 61% for users of combined hormonal contraceptives. Thus, maintenance of lactation itself was notably higher among women using single-ingredient pills containing only progestogen compared to those using combined hormonal contraceptives [26].

Following oral administration, desogestrel is rapidly absorbed and metabolized into etonogestrel. In breastfeeding women, etonogestrel is secreted into milk at a ratio of 0.37–0.55 milk/plasma. Based on these findings, an infant may ingest 0.01–0.05 mg of etonogestrel with an average daily milk intake of 150 mL/kg/day [27]. The available observations of infants breastfed for 7 months whose mothers (*n* = 42) started 75 μg DSG and of those (*n* = 40) whose mothers used a copper-releasing intrauterine therapeutic system from day 28 to 56 postpartum showed no significant differences in length, weight, or head circumference after 1, 4, and 7 cycles of treatment. Although breast enlargement (*n* = 2) and increased sweating (*n* = 1) were noted during the initial cycles of oral contraceptive intake, these effects were no longer there after 1.5 and 2.5 years, and there were no differences in growth scores and physical and psychomotor development among children. However, one participant withdrew from the study due to reduced lactation in the DSG group. Notably, no significant variations were observed in the amount of milk produced, triglycerides, and lactose in milk [27]. Also, in a study by Dutta et al. [28], no differences were found in the amount of milk produced, growth, and the development of infants between the DSG (*n* = 200) and PLACEBO (*n* = 200) groups for 6 months, starting at 6 weeks postpartum [28]. Dilbaz et al. [29] also reported no negative effects of DSG on breastfeeding among 4964 women (DSG from day 21 postpartum). During follow-up, the percentage of women breastfeeding at 3, 6, and 9 months postpartum was 68.4%, 54.8%, and 58.5%, respectively [29]. On the other hand, in a study by Janus et al. [30], after 28 days of DSG use (3 months postpartum), contraception was discontinued due to the onset of scrotal hair in the newborn (resolved at 11 months) [30].

Orally administered drospirenone (DRSP) is rapidly and almost completely absorbed, with 95–97% binding to plasma albumin [31]. Only small amounts of drospirenone pass into human milk, with the daily amount consumed by a baby being less than 1% of the mother’s dose. Melk et al. [32] observed that an average of 18.13% of DRSP permeates into breast milk, and the total amount of DRSP secreted with milk averages 4478 ng/24 h, which is equivalent to 0.11% of the daily dose taken by the mother. Given this minimal transfer into human milk at recommended doses of 4 mg/day, no adverse effects of drospirenone on breastfed newborns/infants are anticipated [32]. Blode et al. [33] studied the pharmacokinetic properties of DRSP in the serum and milk of breastfeeding women who received a single oral dose of 3 mg DRSP with 30 μg ethinylestradiol (EE). The average concentration of DRS in breast milk 24 h after administration was 3.7 ± 1.9 ng/mL, with an average transfer of 635 ng over 24 h (approximately 0.02% of the maternal dose). The authors estimated that the daily dose reaching the infant through breast milk was about 3 μg (based on the average concentration of the drug in breast milk over 24 h and assuming a daily intake of about 800 mL of breast milk) [33]. The pharmacokinetic parameters, including the permeation of drospirenone into breast milk following repeated oral administration of 4 mg DRSP and 3 mg DRSP with 30 μg EE, remain consistent in both serum and breast milk, indicating that lactation has no effect on the pharmacokinetic parameters of the drug [34]. Thus, no adverse effects on neonates and/or breastfed infants are expected with 4 mg drospirenone. Notably, the 24 + 4 day regimen of 4 mg drospirenone demonstrates high safety, and a high acceptance rate that is documented with a low incidence of treatment discontinuation due to unacceptable changes in bleeding profiles [22,24,35]. DRSP (*n* = 71) used postpartum statistically reduces the risk of postpartum depression compared to the control group (*n* = 78), as assessed by the Edinburgh Postpartum Depression Scale at 12 and 24 weeks postpartum, particularly in women with individual steroid sensitivity [36].

While single-ingredient contraception offers numerous advantages, it can also cause side effects and adverse reactions that are worth considering, especially in breastfeeding women. When using single-ingredient contraception, the following can occur with varying frequency: mood changes, weight fluctuations, breast pain, headaches, migraines, decreased libido, skin issues, and nausea. Irregular bleeding or changes in bleeding profile, during single-ingredient contraceptive use, are side effects frequently reported in clinical trials; however, they are generally well tolerated [22,37,38,39].

### 3.2. Scheduled Single-Ingredient Hormonal Contraception in the Form of a Subcutaneous Implant or an Intrauterine Therapeutic System

Long-term hormonal contraception, such as the subcutaneous implant (containing 68 mg micronized etonogestrel) or an intrauterine therapeutic system (with levonorgestrel) has many supporters among breastfeeding women due to its convenience, eliminating the need for regular oral pill intake. Depot medroxyprogesterone acetate (DMPA) in a dose of 150 mg every 12 weeks is a highly effective contraceptive agent. The World Health Organization recommends that injectable depot medroxyprogesterone acetate should not be used before 6 weeks postpartum [15].

#### 3.2.1. Effectiveness

The Pearl index for the subcutaneous etonogestrel implant ranges from 0 to 0.3 [22]. For intrauterine therapeutic systems containing 52 mg of levonorgestrel, the Pearl index ranges from 0.09 to 0.11, while with 13.5 mg of levonorgestrel, the Pearl index reaches 0.33 [22].

#### 3.2.2. Effects of Contraception on Breastfeeding and the Newborn Baby

The etonogestrel implant does not compromise breast milk quality; however, small amounts of etonogestrel are secreted in breast milk. Based on published data, the risk of lactation inhibition with the etonogestrel implant is approximately 0.9% [40]. Assuming a daily milk intake of 150 mL/kg, infants consume an average daily dose of about 19.9 ng/kg/day of etonogestrel after 1 month, decreasing to 15.1 ng/kg/day after 2 months and 10.5 ng/kg/day after 4 months of implant use. Thus, the concentration of etonogestrel in breast milk during lactation decreases with the duration of use of the aforementioned contraceptive method [41]. In a study by Vricella et al. [42], a subcutaneous implant with etonogestrel was used in 42 women between 28 and 56 days postpartum. Compared to infants of 38 mothers who received a nonhormonal IUD, there were no statistically significant differences in growth rates between the groups, except for a slightly greater weight gain in male infants and occurrences of respiratory and skin diseases in infants of women with implants [42]. Additionally, no disparities were observed in milk volume, lactose, protein, and fat content [41] or lactation duration [43] between women using an implant or a nonhormonal IUD. A randomized prospective study by Carmo et al. [44] found no significant differences in breastfeeding rates and newborn growth between mothers receiving an etonogestrel implant within 48 h (*n* = 50) of delivery or 6 weeks (*n* = 50) after delivery during the first year of life [44]. Similarly, placing an etonogestrel implant within 48 h after delivery (*n* = 12) did not affect the milk intake or weight gain of the newborns at follow-up (48 h and 29 days postpartum) [45].

The levonorgestrel-releasing intrauterine therapeutic system (IUDs that release either 10 mcg or 30 mcg of levonorgestrel daily placed at 6 weeks postpartum) does not compromise the quantity or quality of breast milk. Only minimal amounts of progestogen (approximately 0.1% of the levonorgestrel dose) are transferred into the milk of nursing women [46]. In a prospective, nonrandomized study conducted by Bahamondes et al. [47], the effects of four contraceptive methods on milk production were compared. Initiated on the 42nd postpartum day, the study included a binary combination formulation of ethinylestradiol 30 μg with levonorgestrel 150 μg, an etonogestrel implant (68 mg), a levonorgestrel-releasing intrauterine system (52 mg), and a copper IUD. The average infant milk intake, weight and height gain, number of breastfeeding episodes, daily diaper changes, and duration of exclusive breastfeeding were similar across all four groups. Thus, no statistical differences were observed in milk intake or infant development between the methods from day 42 to day 63 of contraceptive use [47]. Furthermore, when comparing the percentage of breastfeeding women who received either an etonogestrel implant (*n* = 28) or a levonorgestrel implant (*n* = 112) immediately after delivery, no difference was observed in the rate of exclusive breastfeeding after 6 months between the groups or in the continuation of breastfeeding for up to 2 years [48]. IUDs that released 10 mcg (*n* = 30) or 30 mcg (*n* = 40) of levonorgestrel daily were placed at 6 weeks postpartum. Copper-releasing intrauterine devices were used as controls (*n* = 40). No differences in height, weight, development, respiratory infections, or blood chemistry were observed in infants up to 12 months of age between the levonorgestrel and copper IUD groups. The rate of breastfeeding discontinuation was higher with the levonorgestrel groups than in the copper IUD group at 75 days, but not at other times [46]. In a study of 320 lactating women who had an intrauterine device containing 52 mcg of levonorgestrel (*n* = 163) vs. an intrauterine device containing Cu T380A (*n* = 157). follow-up of infants for 1 year showed no differences in growth and development or breastfeeding duration [49].

Long-term hormonal contraception containing only progestogen during lactation offers a convenient and effective contraceptive option (eliminating the need for regular oral pill intake), while potentially safeguarding against or at least not exacerbating bone mineral density loss during lactation [50,51]. However, intramuscular systems should be used with caution, especially in women with congenital or valvular heart defects and those with diabetes mellitus.

### 3.3. On-Demand Emergency Single-Ingredient Hormonal Contraception

In lactating women without other contraindications, emergency contraception following intercourse in the form of oral pills (containing 1.5 mg levonorgestrel or 30 mg ulipristal acetate) is the preferred method. To ensure the safety of this contraceptive approach, some authors suggest refraining from breastfeeding for at least 8 h after using 1.5 mg levonorgestrel [52], while some others suggest refraining for 3–4 h [53]. Conversely, some authors do not specify the need to interrupt breastfeeding after levonorgestrel intake [54]. After taking ulipristal acetate, the manufacturer advises against breastfeeding for 7 days [55], whereas the LactMed database recommends avoiding breastfeeding for 24 h after administration [55,56,57]. A 7 day avoidance of breast feeding is more harmful in the long run for both mother and baby if breast milk is significantly reduced because the baby is not being breast fed. The above variations are quite important, and it seems reasonable to present them to mothers, allowing them to decide how to proceed with breastfeeding. Should a mother opt to temporarily suspend breastfeeding due to concerns about her child’s health and then wish to resume breastfeeding after a selected period, it is necessary to outline lactation management principles and present alternative feeding methods to mitigate the risk of breastfeeding difficulties related to disorders of the sucking function of the breast [58,59].

#### 3.3.1. Effectiveness

In the case of emergency contraception, the number of pregnancies after a single dose of 1500 μg levonorgestrel (taken within 72 h of unprotected intercourse) was 35 (2.2%) among 1625 women using this method, compared to 22 (1.4%) pregnancies among 1617 using 30 mg ulipristal acetate [60]. IUDs are a highly effective method of emergency contraception, as 99.86% of users overall did not become pregnant after unprotected intercourse when an IUD was inserted post-coitally [61].

#### 3.3.2. Effects of Contraception on Breastfeeding and the Newborn Baby

In the sole available study, milk samples were collected from 12 breastfeeding women after presumed administration of ulipristal (no dose specified, but likely 30 mg) at 24 h intervals to measure the concentration of ulipristal acetate and its active metabolite. Based on these findings, it was estimated that a fully breastfed infant would ingest approximately 4.1 mcg/kg of the drug along with its active metabolite on the first day, increasing to a total of 5.2 mcg/kg over 5 days [62]. Levonorgestrel, when used for emergency contraception via oral administration, is rapidly and almost completely absorbed and passes into breast milk. An observational study by Polakov–Farkash [54] spanning from January 2005 to January 2010 compared the effects of levonorgestrel emergency contraception to ethinyldiol acetate or desogestrel on the incidence of adverse side effects during lactation for both mother and child. The results of the study confirmed the safety of using levonorgestrel as an emergency contraceptive during lactation, without the need to interrupt breastfeeding, as reductions in lactation were rare and similar between the study and control groups. Adverse effects in infants were minimal (0 out of 72 infants versus 2 out of 72 infants, *p* = 0.5 in the study group versus the control group) [54]. Another cohort study found no statistically significant differences in infants following maternal use of LNG as an emergency contraceptive (in terms of weight, length, head circumference, chest circumference, and mid-arm circumference at 3 and 6 months postpartum, as well as Denver Developmental Screening Test scores at 6 months postpartum [63]. A single administration of the 1.5 mg LNS pill did not objectively affect the health and development of nursing infants, nor did it subjectively affect the amount of milk secreted. As of this update, no relevant published information has been found regarding the effects on breastfed infants, lactation, and breast milk following ulipristal acetate use [54,63,64,65].

### 3.4. Progestogen Hormonal Contraceptive Methods during Lactation—Clinical and Practice 

The table below presents the practical use of progestogen hormonal contraceptive methods during lactation (Table 2). This is a useful summary of everyday use by a breastfeeding patient.

## 4. Conclusions

Based on published scientific reports, progestogen hormonal contraceptives can be considered a relatively safe solution for women desiring to continue feeding their infant with their milk while using hormonal contraception. It is important to seek guidance on selecting the best contraception method based on the latest medical knowledge, tailored to the individual needs and clinical circumstances of each woman and place of residence. A woman should always be informed of the potential risks of such a treatment and then allowed to make her own decision based on the knowledge received from a specialist.

## Figures and Tables

**Table 1:** Search strategy on electronic databases and results.

Database	Search Strategy	Results
PubMed (MEDLINE) Cochrane Database	1.1. “lactation” and “oral contraception” with focus on options like: “drospirenone”, “4 mg of drospirenone” or “desogestrel”, “75 μg of desogestrel” 1.2. “breastfeeding” and “oral contraception” with focus on options like: “drospirenone”, “4 mg drospirenone” or “desogestrel”, “75 μg desogestrel”	*ad. 1.1.* with “drospirenone” (*n* = 5), with “4 mg drospirenone” (*n* = 4) *or * with “desogestrel” (*n* = 16), with “75 μg desogestrel” (*n* = 0) *ad. 1.2.* with “drospirenone” (*n* = 3), with “4 mg drospirenone” (*n* = 3) *or * with “desogestrel” (*n* = 13), with “75 μg desogestrel” (*n* = 0)
	2.1. “lactation” with focus on options like: “subcutaneous etonogestrel implant”, “etonogestrel implant” or “levonorgestrel-releasing intrauterine system” 2.2. “breastfeeding” with focus on options like: “subcutaneous etonogestrel implant”, “etonogestrel implant” or “levonorgestrel-releasing intrauterine system”	*ad. 2.1.* with “subcutaneous etonogestrel implant” (*n* = 1), with “etonogestrel implant” (*n* = 28) or with “levonorgestrel-releasing intrauterine system” (*n* = 16) *ad. 2.2.* with “subcutaneous etonogestrel implant” (*n* = 1), with “etonogestrel implant” (*n* = 30) or with “levonorgestrel-releasing intrauterine system” (*n* = 17)
	3.1. “lactation” and “oral contraception” with “emergency contraception”, with focus on options like: “levonorgestrel”, “1.5 mg levonorgestrel” or “ulipristal acetate”, “30 mg ulipristal acetate” 3.2. “breastfeeding” and “oral contraception” with “emergency contraception”, with focus on options like: “levonorgestrel”, “1.5 mg levonorgestrel” or “ulipristal acetate”, “30 mg ulipristal acetate”	*ad. 3.1.* with “levonorgestrel” (*n* = 9), with “1.5 mg levonorgestrel” (*n* = 0) or with “30 mg ulipristal acetate” (*n* = 0), with “ulipristal acetate” (*n* = 2) *ad. 3.2.* with “levonorgestrel” (*n* = 10), with “1.5 mg levonorgestrel” (*n* = 0) or with “30 mg ulipristal acetate” (*n* = 0), with “ulipristal acetate” (*n* = 3)

**Table 2:** Progestogen hormonal contraceptive methods during lactation—clinical and practice.

Scheduled Contraception	Use *	Effects on Breastfeeding	Safety of Use in the Newborn
75 μg desogestrel —1 × 1 pill	- can be started on any day after delivery - without menstruation: between the 21st and 28th days does not necessitate additional mechanical contraceptive methods. If started later, pregnancy should be ruled out, and an additional mechanical contraceptive method should be used for the first week. - with menstruation: take the first pill on the 1st day of menstrual bleeding; when using between the 2nd and 5th day of the cycle, it is recommended to use an additional mechanical contraception method for the first 7 days of this cycle.	Desogestrel is metabolized to the active metabolite—etonogestrel−0.01 to 0.05 μg of etonogestrel per kg bw/day can be taken in by an infant (considering an estimated milk intake of 150 mL/kg bw/day) [27].	It does not seem to affect the production or quality of milk secretion, although, in the postpartum period, a reduction in milk production was noted but not very often (…) [27,28,29,30]
4 mg drospirenone —1 × 1 pill		- the amount of drospirenone that the baby takes up through milk is <1% of the dosage taken by the mother [31,32].	(…) at therapeutic doses, it is not expected to affect newborns and/or breastfed infants (…) and can be used during lactation. DRS is a progestogen analog of spironolactone. It has anti-mineralocorticoid and anti-androgenic properties. The amounts in milk are minimal and no adverse effects are anticipated on the breastfed infant or milk supply [31,32,33,34,35,36,37,38,39].
Subcutaneous implant (68 mg of micronized etonogestrel)	Nonbreastfeeding women: insertion on the 21st to 28th day after childbirth does not require additional contraceptive methods. If insertion occurs later than 28 days after delivery, use barrier methods for an additional 7 days. If sexual intercourse has taken place, pregnancy should be ruled out. Breastfeeding: insertion 4 weeks after childbirth along with the use of a barrier method for 7 days. If sexual intercourse has taken place, pregnancy should be ruled out.	- Considering a milk intake of 150 mL/kg bw, infants may ingest an average of 27 ng/kg bw/day after one month, which corresponds to approximately 2.2% of the maternal daily dose, calculated relative to body weight, and about 0.2% of the estimated absolute maternal daily dose. The concentration of etonogestrel in breast milk during breastfeeding decreases over time [40,41].	It does not affect the secretion or quality of breast milk (...), although the child’s growth and development should be carefully monitored. It does not affect the secretion and quality of breast milk and has no effect on newborn development [42,43,44,45].
The levonorgestrel-releasing intrauterine system (LNG IUS)	Nonbreastfeeding women: postpartum insertion until uterine involution is complete, usually occurring 6 weeks after delivery. If involution is delayed, consider waiting until 12 weeks after delivery. Breastfeeding: “the use of contraceptives containing only progestogens, initiated from the 6th week after delivery, has no harmful effect on the growth or development of the child (...) and the quantity or quality of breast milk (...)”.	- About 0.1% of the dose may pass through milk into the baby’s body [46].	It has no harmful effects on the growth and development of the child (...) nor on the quantity or quality of breast milk. It does not affect the secretion and quality of breast milk and has no effect on the development of the neonate [47,48,49,50].
**Emergency Contraception**	**Use**	**Effects on Breastfeeding**	**Safety of Use in the Newborn**
with 1.5 mg LNG—1 × 1 pill	Nonbreastfeeding women: Take 1 pill as soon as possible, preferably within 12 h and no later than 72 h after unprotected sex. Breastfeeding: some authors suggest refraining from breastfeeding for at least 8 h after using 1.5 mg levonorgestrel [51], while some others suggest refraining for 3–4 h [52]. During this time, pump mechanically and discard milk to stimulate lactation. Conversely, some authors do not specify the need to interrupt breastfeeding after levonorgestrel intake [53].	- About 0.1% of the dosage can enter a breastfed infant’s body through milk [53].	Permeates into human milk. “A single application of 1.5 mg LNS tablet does not objectively affect the health and development of the breastfed child, nor does it subjectively affect the amount of milk secreted” [61].
30 mg ulipristal acetate —1 × 1 pill	Nonbreastfeeding women: Take 1 pill as soon as possible, no later than 120 h (5 days) after unprotected sex in the event of contraceptive method failure. Breastfeeding: as above; it is recommended to avoid breastfeeding for one week (7 days) [54], whereas the LactMed database recommends avoiding breastfeeding for 24 h after administration [54,55].	- Based on these findings, it was estimated that a fully breastfed infant would ingest approximately 4.1 mcg/kg of the drug along with its active metabolite on the first day, increasing to a total of 5.2 mcg/kg over 5 days [60].	Effects on neonates and/or infants have not been studied. Risk to the breastfed infant cannot be ruled out. Effects in breastfed infants, lactation, and breast milk—no relevant published information was found as of the date of this update [LactMed].

* Menstruation may occur during breastfeeding or partial breastfeeding. Therefore, the table includes menstruating and non-menstruating patients during breastfeeding.

## Data Availability

Not applicable.
